# Evidence of Authorship on Messages in Facilitated Communication: A Case Report Using Accelerometry

**DOI:** 10.3389/fpsyt.2020.543385

**Published:** 2021-01-14

**Authors:** Patrick Faure, Thierry Legou, Bruno Gepner

**Affiliations:** ^1^Laboratoire Parole et Langage (LPL), CNRS, Aix-Marseille Université, Aix-en-Provence, France; ^2^Laboratoire Parole et Langage (LPL), CNRS, Institute of Language Communication and the Brain (ILCB), Aix-Marseille Université, Aix-en-Provence, France; ^3^Institut de Neurophysiopathologie (INP), CNRS UMR 7051, Aix-Marseille Université, Marseille, France

**Keywords:** facilitated communication, autism spectrum disorder, motor disorders, physical support, accelerometry, authorship, coproduction, validity

## Abstract

*Facilitated communication* (FC) belongs to augmentative and alternative methods of communication. Currently, FC is very rarely and unofficially used with people suffering from verbal/communicative disorders or neurodevelopmental disorders such as intellectual deficiency or autism spectrum disorder (ASD). FC consists of physical support exerted by a *facilitator* at the hand/wrist/forearm/elbow of a patient/participant, aimed at helping him/her to point at pictures/words, and sometimes to type letters/words on a keyboard. Given most of (but not all) validation studies using control procedures failed to confirm that ASD participants themselves were authoring the messages via FC, this method has been massively disputed and rejected. However, firm and definitive conclusions for/against the validity of FC requires more robust demonstrations, particularly when considering the motor participation of both protagonists. We present here a case report investigating the motor contribution of both protagonists during a typing process using the non-invasive technique of accelerometry. A 17-year-old boy diagnosed with congenital deafness, ASD, and developmental delay, and his facilitator, were equipped with small accelerometers fixed on their index finger, aimed at transforming index acceleration along the three spatial axes into electric signals. Typing on a PC keyboard was performed under three support conditions: hand support, forearm support, elbow support, plus a solo-typing condition. Accelerometric signals and video data were recorded during four FC sessions. We measured and compared the typing speed, the number/percentage of acceleration peaks produced by the participant or by the facilitator first, and those of “signal under detection threshold” in the facilitator, the time offset between acceleration peaks of both protagonists, and the difference of the amount of acceleration between them, across the different support conditions. Results indicate that in the hand support, most of the time, acceleration motions of the participant's index finger preceded those of the facilitator's index finger. Then, the more distal the physical support (i.e., farer from the participant's hand), the slower the speed of typing, the higher the percentage of “signal under detection threshold” in the facilitator, the bigger the motor contribution from the participant. Altogether, in all the support conditions, the participant's authorship or, at least, co-authorship on the messages seems warranted. Finally, accelerometry seems relevant to objectivize authorship or co-authorship in FC and delineate various forms of FC.

## Introduction

*Facilitated communication* (FC) is an alternative/augmentative method of communication. In the 1980s and 1990s, it was used with people suffering from various verbal and communication impairments and mental handicaps [intellectual disability or autism spectrum disorder (ASD)]. It consists of a physical support exerted by a facilitator at the hand (hand-over-hand), wrist, forearm, elbow, or shoulder of a patient or “student,” in order to help him/her to point at objects, pictures, pictograms, and even sometimes to type letters and words on a paper board or an electronic keyboard (1).

Following its first use in Australia by Crossley with teenagers suffering from cerebral palsy in the 1970s and 1980s, FC has received huge interest from clinicians and researchers, but also from the media, first because it appeared somehow miraculous, given it seemed to reveal totally unexpected cognitive and expressive abilities in people suffering from various handicaps, such as cerebral palsy, intellectual disability, and ASD (2). Secondly, FC was involved in several prosecutions. For example, through FC a girl with cerebral palsy and severe intellectual disability claimed to leave her institution for mentally handicapped people to study at school and she obtained the permission to do so by the court (3). There were also several cases of allegations of sexual abuse typed through FC in the USA. In this context, authorship of the messages typed during FC by the complainant was assessed using a control procedure called the message passing procedure (MPP). MPP comes from information systems and consists of delivering the same (unmasked condition) vs. different information (masked condition) to the patient/student and his/her facilitator, and to observe what answer is produced. Given responses of the complainant were all wrong (except a few) in the masked condition, and 100% correct in the unmasked one, allegations were not conclusive (4). In the 1990s, an exponential number of studies were conducted to assess the efficacy and reliability of FC, using various uni- or multi-modal settings (MPP, auditory and/or visual inputs, familiar/unfamiliar facilitator, hand support vs. mechanical device, etc.), alongside with (or without) control procedures (e.g., pre- and post-tests, analysis of audio/video recorded sessions, conditions counterbalanced, etc.) [(5–7) for reviews]. The vast majority of studies using control procedures reached similar or converging general conclusions, i.e., responses of the patients/students were mostly influenced (consciously or unconsciously) by the facilitator, and FC was generally not efficient as a communication or educational tool. As a consequence, FC was—almost—unanimously rejected by the scientific community, and it is currently used unofficially.

In contrast, very few studies using control procedures revealed in some cases that the patients themselves were authoring the words typed via FC [e.g., (8–10)]. Some studies using MPP suggested that FC may have the potential for developing academic skills in some students [e.g., (11)], or for enhancing communication in a few others [e.g., (12)]. Another study demonstrated that participants with ASD performed as well as typically developing participants of the same age in theory of mind and pragmatic tasks when answering via FC (13). There was also minimal evidence of the validity of FC in some participants with intellectual disability [e.g., (14)], particularly in the context of the naturalistic approach of FC (15).

Moreover, as observed by Mostert (5), most of the studies investigating the validity of FC, either those supporting FC or those refuting it, and even those using uni- or multi-modal control procedures, suffer several methodological limitations, including: the number of participants rarely exceeds 10; the experimental setup is often poorly described; and investigations lasted <3 months; moreover when there was more than one participant, precise clinical characteristics of the participants were lacking (e.g., intellectual quotient, verbal comprehension and expression levels, attentional level, reading abilities, etc.).

Altogether, no firm nor definitive conclusion has been reached so far on the validity of FC. As recommended by Mostert (5), “*there is much to be done related to FC, both theoretically and experimentally*” […] “*further attention to facilitation itself might prove useful*,” and “*investigation into differing intensities of facilitator support (e.g., full support at wrist, support only by touching, no support) and the potential for facilitator influence should be more closely investigated*” (p. 311).

Following the recommendation of Mostert (5), we sought in the present study to measure more accurately and robustly, by the means of *accelerometry*, the respective motor contribution of a facilitator and a patient while typing letters/words during FC sessions. Accelerometry is a non-invasive technique that uses an *accelerometer* (a sensor) to transform acceleration along the three *x, y*, and *z* spatial axes into electric signals.

By attaching an accelerometer to the index finger of both a patient with ASD and associated disorders, and his facilitator, we wished to monitor the course of some fine motor events produced by each of the protagonists at the index level during the FC process, particularly the speed of typing, and the temporal course and the amount of acceleration signals produced during keystrokes. We also wished to measure the variations of these parameters according to the location of physical support exerted by the facilitator during the FC process, i.e., from a proximal support (hand support) to an intermediate support (forearm support) and to a distal one (elbow support), plus a solo typing condition. We therefore hypothesized that accelerometry might enable us to objectivize the respective motor contribution of a patient and his facilitator during FC, and crucially, to answer the question of who, among the two protagonists, produces acceleration signals first. Answering this question might produce evidence of the patient's and/or the facilitator's authorship on the messages produced during FC.

## Materials and Methods

This research has been approved by the Ethics Committee of Aix-Marseille University (France).

### Protagonists

#### Participant Presentation

The participant named B.L. is a 17 year-old boy. During the first trimester of pregnancy, his mother had been infected by a cytomegalovirus (CMV). As a consequence, at birth he suffered a thrombocytopenia purpura leading to a diffuse cerebral hematoma, which was resorbed after several weeks. Also due to CMV infection, B.L. was diagnosed with a profound and bilateral deafness at 6 months of age. He benefited from a late cochlear implant (at age 15), after which he could only hear very few sounds.

B.L. also presented a general developmental delay (see below and Table 1). And, at the age of three, he was diagnosed with an infantile autism according to the ICD-10 (16) criteria.

**Table 1:** Clinical characteristics of participant B.L.

**Variable**	**Developmental and cognitive domains**	**Age (year)**	**Score**
**Chronological age**		17	
**CARS**			36
**VABS**	Verbal comprehension	7	
	Verbal expression	–	
	Autonomy	8	
	Motricity	7	
	Socialization	6	
**Early symptoms**	In the first year, excessively calm and quiet, no babbling, no crying, sleep disorders. Diagnosed with severe and bilateral deafness at six months of age. Delayed motor development (walking at 27 months).		
**Current symptoms**	No verbal expression, some atypical vocal sounds, very poor non-verbal communication (gaze, gestures), some appropriate but poor communication in his areas of interest through gestural imitation and atypical mimicries, poor social interaction, lack of social initiative, and restricted and stereotyped interests. Lack of motor anticipation and initiative, general slowness, some repetitive or disorganized movements, clumsiness, and handwriting difficulties (macrography, slowness).		
**Past occupations**	He received a scholarship associating French sign language, pictograms, and words for the past 10 years.		
**Communication and lexical assessment**	Poor communication with pointing, few uses of sign language, pictures, and drawings. He recognizes some simple words, can copy some words and sentences with paper/pen but with a limited comprehension, and types a few words alone (~50 words) belonging to his repertoire of interests (names of relatives, the city where his parents live, names of cartoon characters, movies, and car brands), but no sentences.		

*CARS, childhood autism rating scale; VABS, Vineland adaptive behavior scale; FC, facilitated communication*.

Since the age of four, B.L. has been living in an institute for children with hearing disorders and other associated handicaps.

In the context of the present research, an expert clinician confirmed the clinical diagnosis of autism spectrum disorder of moderate severity, according to a careful clinical examination, a full case history reported by his parents, and reports from professionals of the institute where he is living (particularly his usual teacher, also being his facilitator, see below), and according to the DSM-5 criteria (17) for ASD and the CARS (18) assessment. Developmental scores on the Vineland adaptive behavior scale (19) performed with the parents and professionals, as well as some lexical assessments (see Table 1), confirmed his associated moderate general developmental delay (developmental quotient around 40). He is mute and communicates mostly by pictures, drawings, and some handwritten or typed words. His full clinical description is presented in Table 1.

When he was four, his parents learned French sign language, and used to communicate with him by this means, but B.L., although understanding basic French sign language, very rarely uses it.

His parents gave fully informed consent for his participation in the present study.

#### Facilitator Presentation

The facilitator, named M.T., is an experienced teacher for teenagers with various handicaps (deafness, ASD, and intellectual deficiency). During the last 5 years, she has communicated with B.L. with French sign language, pictograms, and handwritten or typed words on a PC. She has also received complete training in FC and is certified in FC. She started to use FC with B.L. 3 years prior the experiment, exclusively by hand support.

For the purpose of the experiment, both B.L. and M.T. agreed to wear accelerometers and to vary the type of support during the sessions of FC (see below).

### Materials

#### Accelerometer

An accelerometer is a small sensor delivering an electrical signal proportional to its acceleration. The accelerometer is set on the device under test (DUT), and when the DUT accelerates, the sensor indicates the magnitude and the sign of the acceleration along three orthogonal *x, y*, and *z* (i.e., lateral, vertical, and anteroposterior) axes.

The accelerometer provides information about the motion of the DUT, i.e., its acceleration or speed variation, but can also provide information about forces applied to it, thanks to Newton's second law of motion *F* = m/a [with *F*: sum of the forces applied to the DUT (in N); *m*: mass (in kg), and *a*: acceleration (in m/s^2^)].

The setup used is based on two three-axes accelerometers (type ADXL335, weighing ± 1.5 g) read by an eight-channel digital audio recorder (type R24, see below) connected to a laptop and controlled via Audacity®, an audio-digital editing software (see below).

We used two accelerometers. One accelerometer was fastened using a “hook-and-loop” ribbon (Velcro®) on the second phalanx of the index finger of the right hand of B.L. The wire connection was attached to the forearm using another Velcro® strip in order to avoid any perturbation to hand motion during the session. Another accelerometer was similarly attached to the facilitator's left index finger, with the wire connection attached to her left forearm.

The sampling frequency used (44.1 kHz) led to a time measurement accuracy that was far better than the one needed to evaluate each finger acceleration over time.

#### Zoom R24® Multitrack Audio Recorder

A Zoom R24® multitrack audio recorder can synchronously digitize several analog signals at a sufficient sampling rate. The three output signals delivered by each sensor ADXL335 (giving information on the acceleration along the three *x, y*, and *z* axes) are amplified and connected to the R24 sound card inputs.

The R24 multitrack recorder module is originally devoted to audio signal digitization but its use in our study was an efficient and easily duplicable way for digitizing signals from both accelerometers.

We characterized the band pass of the R24 sound card (which was not specified in the datasheet) to be sure that it was compatible with low frequency signals that would result from low accelerations. Using a frequency generator, we checked that the cut-off frequency of the input high-pass filter of the R24 sound card was low enough to monitor the acceleration of the index fingers during the use of a keyboard and would allow us to monitor key presses and release.

#### Control and Visualization Software

To control (start and stop) and visualize the analog signal acquisition, we used Audacity® (Version 1.3 Beta Unicode) implemented on a PC. It allowed us to display seven channels (2 × 3 for accelerometers plus one dedicated to synchronization with the video data, see below).

**Figure 1:**
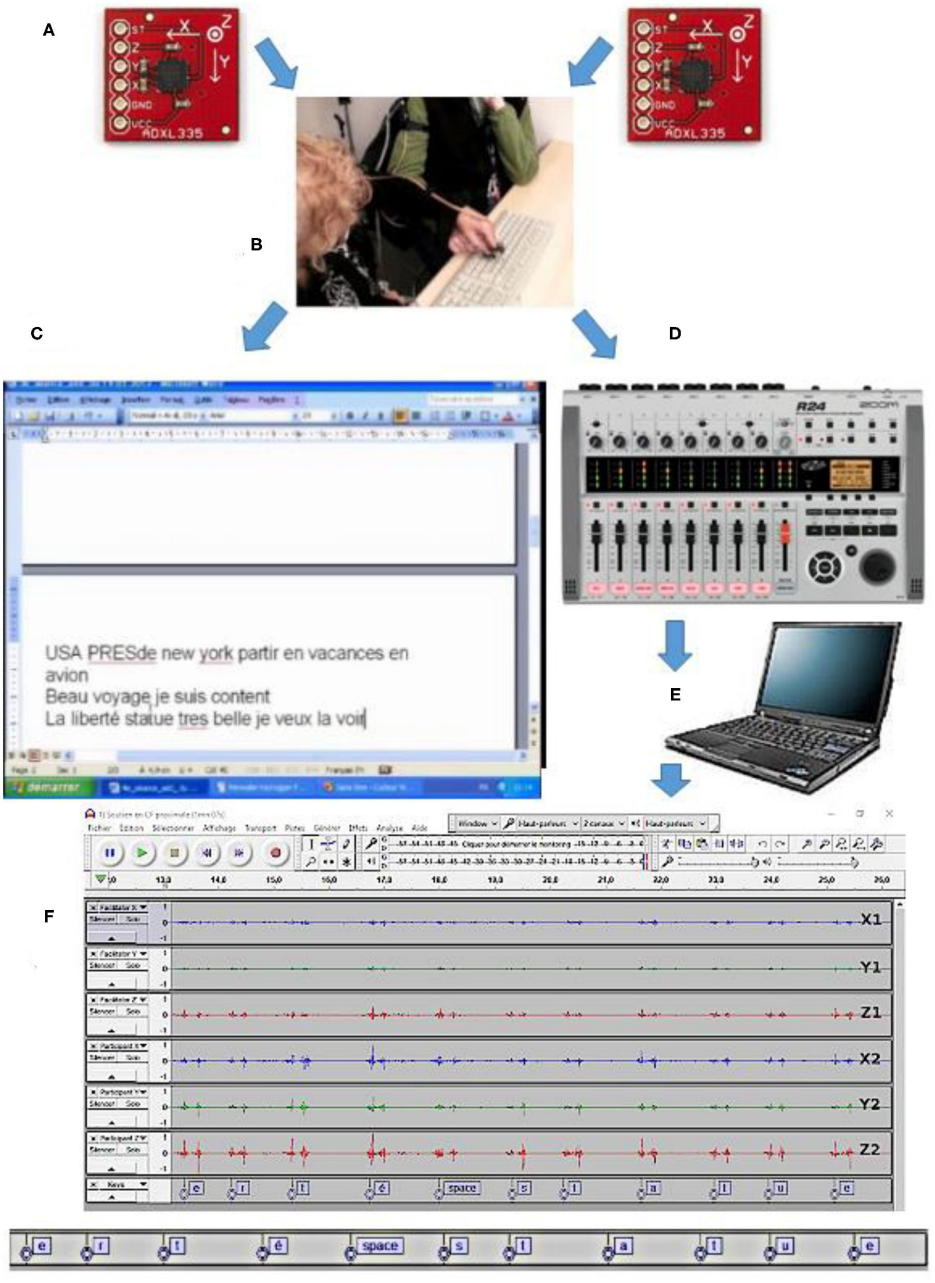
Materials: **(A)** two ADXL335 triple axes accelerometers; **(B)** photograph showing the two accelerometers attached to the index fingers of both the participant and his facilitator; **(C)** PC screen showing typed words and sentences; **(D)** Zoom R24® digital multitrack recorder; **(E)** PC for Audacity® display; **(F)** accelerometric signals displayed with Audacity (on X1, Y1, and Z1 axes for the facilitator and X2, Y2, and Z2 axes for the participant).

Signals from both accelerometers were displayed simultaneously, i.e., *x*1, *y*1, and *z*1 signals from the accelerometer of the facilitator, and *x*2, *y*2, and *z*2 signals from the accelerometer of the patient (see Figure 1).

Accelerometric data were permanently captured, stored on hard disk, and then post-synchronized (precisely superimposed) with (i) video data of the patient-facilitator interactions and (ii) the permanent screen capture (using Windows Media codec) of the text typed during the FC sessions (see Supplementary Video).

#### Video Apparatus

Two HD cameras (Sanyo® XACTI 1000 HD®) set on tripods were used to capture (and record in HD at 30 frames/s) the faces and hands of patient B.L. and facilitator M.T. during the experimental FC sessions.

#### Audio Player

An audio player (Korg Sound®) delivered a signal (that was recorded on a supplementary channel other than the 3 × 2 channels dedicated to the two accelerometers, see above) used as a “time-code” to precisely post-synchronize the permanent flow of the electric signals (which had a sampling frequency of 44.100 Hz) with the permanent flow of the two cameras (converted in SD at 25 frames/s, i.e., an extremely low frequency of 25 Hz).

#### Other Materials

We used three PCs: one connected to a keyboard (used for typing) to view and record the text; one connected to the R24 multitrack recorder to record and monitor the accelerometric signals via Audacity® and one used for the lexical assessments of B.L. (see Table 1)

Finally, accelerometric and video data were stored on several external hard disks (3To).

The materials and whole setup are illustrated in Figure 1 and Supplementary Video.

#### Metrics

Several terms need to be defined before presenting the specific metrics related to accelerometry.

An acceleration signal corresponds to an acceleration motion leading to or not leading to a keystroke.

The peak of acceleration, or acceleration peak, is the instant when the acceleration signal crosses the detection threshold, and can therefore participate to a keystroke.

A keystroke is the pressure of the index finger of the participant B.L. on a key, leading to a typed letter or a sign (“delete,” “return,” and “space”). When analyzing the temporo-spatial dynamics of a keystroke, this is composed of a variable number of peaks (1–4). Peaks were further analyzed only if they occurred in a keystroke, i.e., if the action occurred in an observation window around the keystroke (500 ms before the keystroke and 1 s after it). Given the relatively small number of trials, all the keystroke detections were made visually (on the accelerometric data).

Four types of parameters associated to keystrokes were analyzed in this study:

1- The typing speed: the number of letters or signs typed by one or both protagonists in a minute. It was measured manually from the two accelerometers' output signals. Typing errors were also detected on the PC screen during the FC sessions and on the video data.
2- The time offset between acceleration peaks of the participant and those of the facilitator: this was measured visually. Peaks were dated by measuring the instant when the acceleration signal “leaves” its baseline level and crosses the detection threshold. From this baseline, mean (μ) and standard deviation (σ) of the signal are extracted, and the peak instant is detected when the signal crosses μ+3σ.
3- The number and percentage of (i) acceleration peaks produced by the participant first (when the first acceleration peak leading to a keystroke is produced by the participant), (ii) acceleration peaks produced by the facilitator first (when the first acceleration peak leading to a keystroke is produced by the facilitator), and (iii) signal under detection threshold in the facilitator, when the facilitator's acceleration signal does not cross μ+3σ.
4- The difference in the amount of acceleration between the participant and facilitator: to measure the amount of acceleration associated to a keystroke, we first calculated the quadratic sum of the acceleration signals along the *x, y*, and *z* axes, for both the participant and facilitator, thus obtaining values that were independent from the spatial orientation of the accelerometer of each participant, but also independent from the relative orientations of the two accelerometers during gestures. Then we calculated the integral of the quadratic sum of acceleration, or IQSA, for both the participant and facilitator, which presented the global amount of acceleration recorded during the gesture associated to a keystroke. Then we subtracted the IQSA of the facilitator by the IQSA of the participant.

### Experimental Procedure

B.L. and the facilitator were seated in a quiet experimental room (at the Laboratoire Parole et Langage). The facilitator was seated on the right side of B.L.

Before starting a session, some motion tests were performed for both the patient and facilitator accelerometers, as well as some synchronization tests between the two accelerometers, in order to check that the whole setup was ready. Then the permanent recording of the six signals from the two sensors, plus the timing signal, as well as the permanent recording from the two video cameras, were started.

Then the FC session was started. B.L. typed letters/words/sentences alone or with the facilitator's support, either spontaneously or in response to proposals or questions asked by the facilitator orally and either through French sign language and/or using handwritten or typed words.

**Figure 2:**
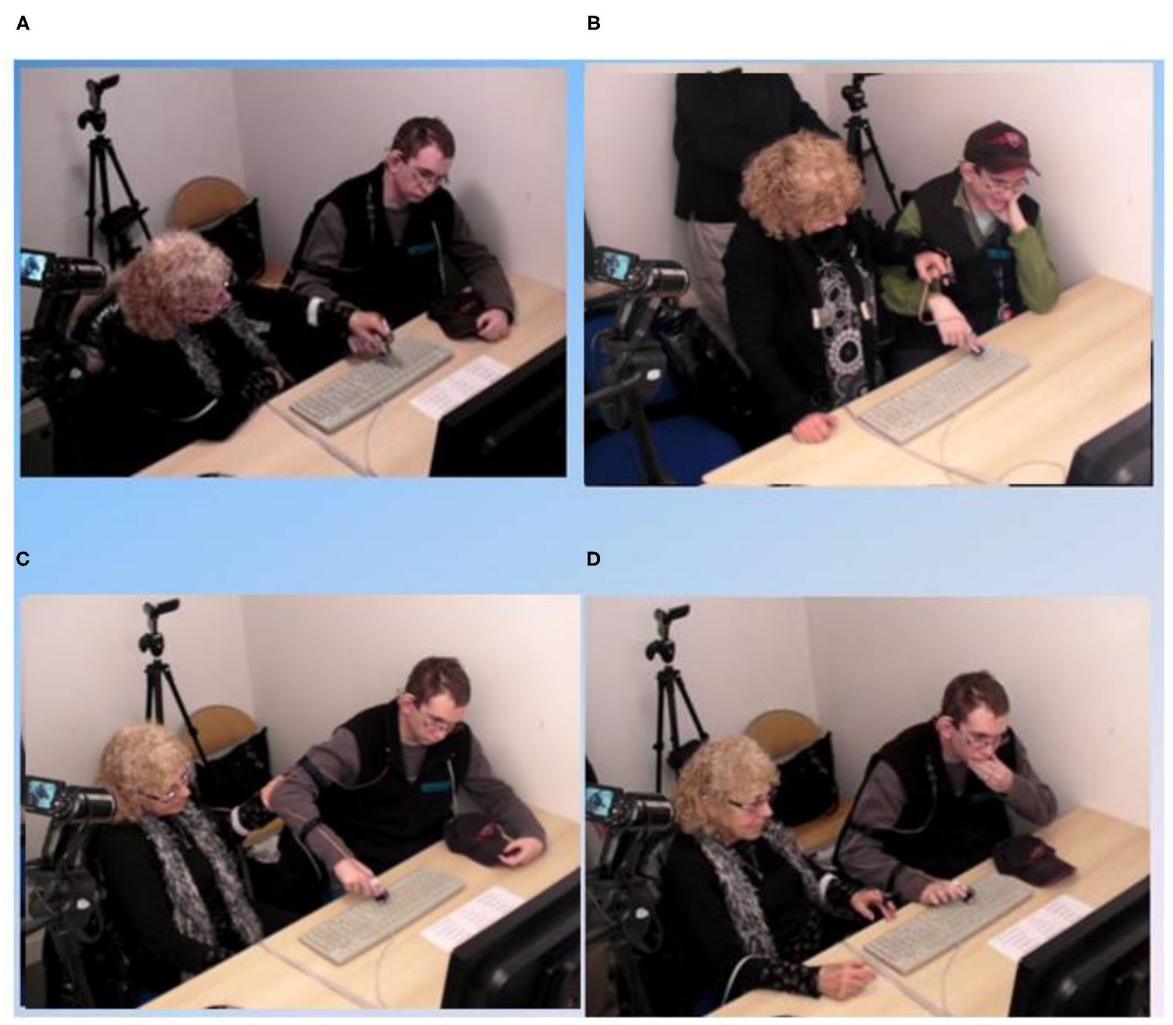
Procedure: The four different typing conditions. **(A)** typing with hand support; **(B)** typing with forearm support; **(C)** typing with elbow support; **(D)** solo typing.

Accelerometric signals and video data were recorded during four FC sessions of about one hour each (including material installation and testing). One session (the last one) could not be filmed properly, therefore it was rejected. During each session, participant B.L. typed on the keyboard under four conditions (as shown in Figure 2 and Video) in a random order that was counterbalanced across sessions:

1) Typing with hand support: the hand of facilitator is above and holds the hand of the participant (i.e., hand-over-hand, index-over-index).
2) Typing with forearm support: the facilitator holds the sleeve of the participant (above his wrist).
3) Typing with elbow support: the facilitator supports the participant's elbow
4) Solo typing: participant B.L. types letters/words alone.

We chose to analyze six windows of accelerometric signals, i.e., one window for each of the three support conditions, and three shorter windows for the solo typing condition (see Supplementary Video), plus the corresponding video and text data, extracted from two among the four FC sessions. They were chosen because they were typical of each of the four typing conditions observed in the whole FC experiment, and also because of the verbal content continuity (e.g., the same topic during hand support and solo typing). These samples are relatively short (hand support: 63 s; forearm support: 57 s; elbow support: 80 s; solo typing: 9 s + 13 s + 16 s = 38 s). However, they comprised a sufficiently important and representative quantity of data to warrant further data analyses.

### Data Analyses

As said above, four types of accelerometric data were further analyzed:

1) The typing speed;
2) The time offset between acceleration peaks of the participant and those of the facilitator;
3) The number/percentage of (i) acceleration peaks produced by the participant first, (ii) acceleration peaks produced by the facilitator first, and (iii) Signal under detection threshold in the facilitator;
4) The difference in the amount of acceleration between both protagonists.

We performed ANOVAs to calculate the interaction between each of these parameters and the typing conditions, and Student's *t-*tests to calculate pairwise comparisons of the results between the three support conditions.

## Results

There are four main results of our study.

First, the typing speed varied upon the typing modality: hand support ~33 hits/min; forearm support ~26 hits/min; elbow support ~15 hits/min; and solo typing ~40 hits/min.

An ANOVA calculating the effect of the support modality onto the typing speed showed a significant difference [*F*_(2, 95)_ = 18.88, *p* < 0.0001]. *T*-tests were then performed to achieve pairwise comparisons of the typing speed between the various support modalities. They revealed that the typing speed was faster in hand support than in forearm and elbow supports, and faster in forearm support than in elbow support (see Table 2). In solo typing, typing speed was faster than in all the support modalities, but the verbal content was more limited to the participant's restricted interests and word repertoire.

**Table 2:** Pairwise comparisons of the typing speed across the different support conditions.

**Pairwise comparisons (Mean, SD)**	***t*-test (two-tailed)**	***p***
Hand (*M* = 1.4275, *SD* = 0.61) vs. forearm (*M* = 2.062, *SD* = 0.85)	*t*_(70)_ = −3.6875	*p* < 0.001
Hand (*M* = 1.4275, *SD* = 0.61) vs. elbow (*M* = 5.40, *SD* = 5.1)	*t*_(68)_ = −5.13	*p* < 0.001
Elbow (*M* = 5.40, *SD* = 5.1) vs. forearm (*M* = 2.062, *SD* = 0.85)	*t*_(52)_ = −3.42	*P* = 0.0012

Moreover, typing errors were more frequent during elbow support than during forearm and hand supports. No typing errors were produced in solo typing.

Second, an ANOVA comparing the time offset between the acceleration peak associated to a keystroke in the participant and in the facilitator across the three support modalities revealed a significant difference [*F*_(2, 150)_ = 26.53, *p* < 0.0001]. *T*-tests performed to achieve pairwise comparisons of the time offset between the acceleration peak from the participant and that from the facilitator across the three support modalities, revealed that this time offset was shorter in the hand condition compared with the other two conditions, and did not differ between the latter two (see the full results in Table 3).

**Table 3:** Pairwise comparisons of the time offset between the acceleration peaks of the participant and those of the facilitator across the different support conditions.

**Pairwise comparisons (Mean, SD)**	***t*-test (two-tailed)**	***p***
Hand (*M* = 0.014, *SD* = 0.169) vs. forearm (*M* = 0.0590, *SD* = 0.0452)	*t*_(137)_ = −9.05	*p* < 0.001
Hand (*M* = 0.014, *SD* = 0.169) vs. elbow (*M* = 0.088, *SD* = 0.058)	*t*_(117)_ = −10.81	*p* < 0.001
Elbow (*M* = 0.088, *SD* = 0.058) vs. forearm (*M* = 0.0590, *SD* = 0.0452)	*t*_(46)_ = −1.9	*p* = 0.0631

**Figure 3:**
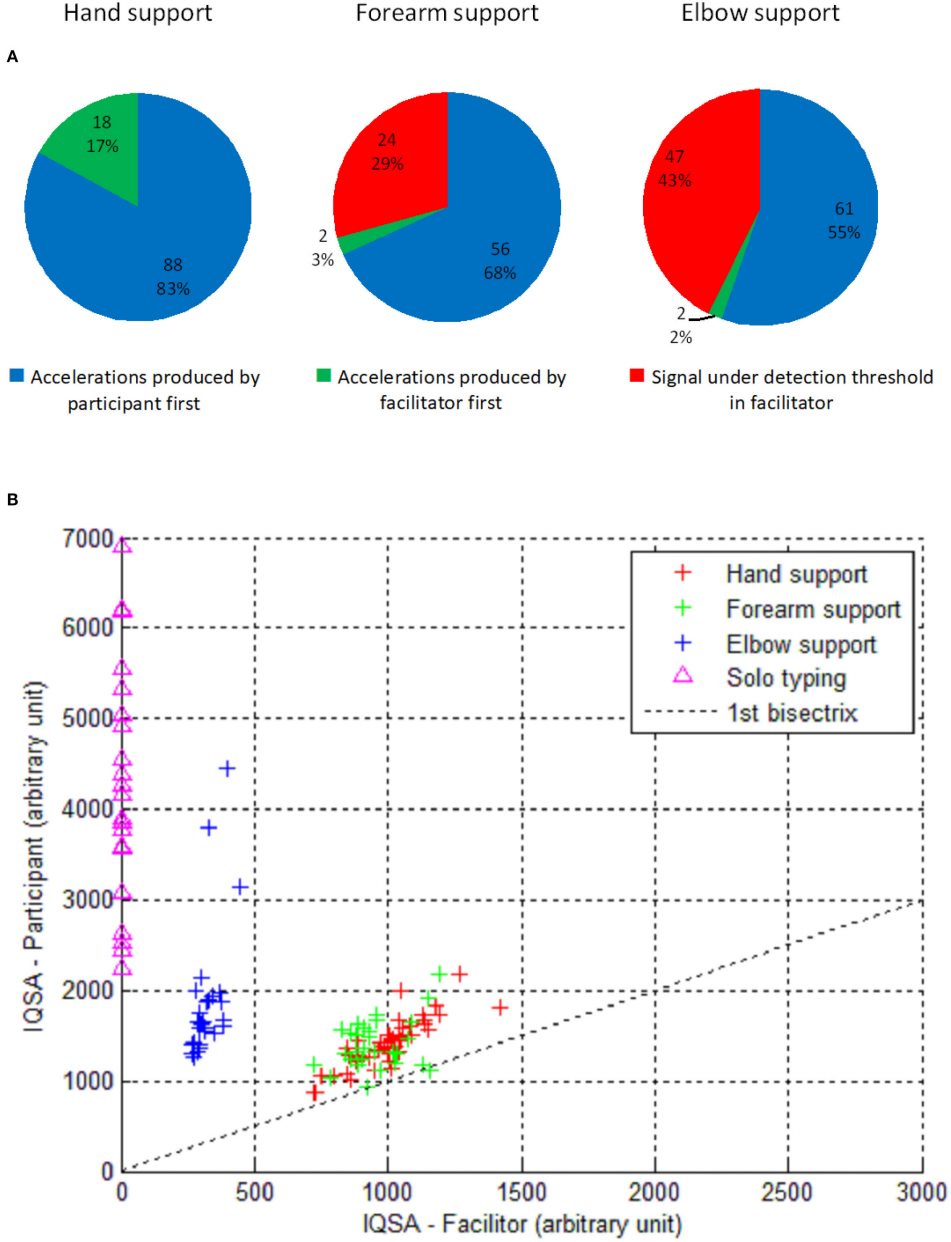
Results: **(A)** pie charts showing the number/percentage of acceleration peaks produced by the participant or facilitator first, and the number/percentage of signal under detection threshold in the facilitator, across the three support conditions. **(B)** Respective amount of accelerations for the participant and facilitator across the different support conditions plus the solo typing condition.

Third, results on the number/percentage of acceleration peaks produced by either the participant or facilitator first, and on signals under detection threshold in the facilitator showed that in the hand support condition, in 83% of cases, the acceleration peak of the participant preceded that of the facilitator, and that the percentage of signals under the detection threshold in the facilitator increased from 0% in the hand support condition to almost 25% in the forearm condition and to almost 50% in the elbow condition. The full results are illustrated in Figure 3A.

Fourth, an ANOVA comparing the differences of IQSA between the participant and facilitator (IQSA participant – IQSA facilitator) across the three support modalities showed a significant difference [*F*_(2, 98)_ = 73.63, *p* < 0.0001]. *T*-tests were performed to achieve pairwise comparisons of the differences of amounts of acceleration between the participant and facilitator in the different support modalities, and revealed that the amount of acceleration was significantly bigger in the participant than in the facilitator in the forearm and elbow support conditions, with no difference between them in the hand support condition. In the participant, the amount of acceleration was bigger in the solo typing condition than in any of the support conditions (see Table 4 and Figure 3B).

**Table 4:** Pairwise comparisons of the difference in the amount of acceleration between the participant and facilitator (IQSA participant—IQSA facilitator) across the different support conditions.

**Pairwise comparisons (Mean, SD)**	***t*-test (two-tailed)**	***p***
Hand (*M* = 408.14; *SD* = 172.21) vs. forearm (*M* = 453.12; *SD* = 256.6)	*t*_(72)_ = 0.903	*p* > 0.2
Hand (*M* = 408.14; *SD* = 172.21) vs. elbow (*M* = 1,556.68; *SD* = 726.25)	*t*_(70)_ = 10.18	*p* < 0.001
Elbow (*M* = 1556.68; *SD* = 726.25) vs. forearm (*M* = 453.12; *SD* = 256.6)	*t*_(54)_ = −7.68	*p* < 0.001

## Discussion

Let us analyze and discuss our results in regard to the main question of authorship on the messages produced during the FC process, i.e., who is typing the messages?

First, in the solo typing condition, given that the participant is typing alone without any physical support, his authorship on the messages is undoubtable. In this condition, the typing speed is faster, and the amount of acceleration is bigger, than in all the three other support modalities, i.e., he types rapidly and vigorously, which is probably due to the fact that the verbal content of the typed words is limited to single words belonging to his restricted interests' and words' repertoires, so that he is used to typing these words. Comparatively, the messages are richer (made of more or less complex sentences) in the three support conditions.

Second, in the hand support condition, we observed that in 83% of cases, the acceleration peak of the participant preceded that of the facilitator, while the acceleration peak of the facilitator preceded that of the participant in only 17% of cases. This result showed that most of the time, the participant was not passively supported or influenced by the facilitator but instead that he contributed actively to motion acceleration toward the letters, and preceded the facilitator, which seems to objectivize his strong contribution to authorship on the messages. Besides, the typing speed was faster in the hand condition than in the other two support conditions, supporting the idea that the hand condition was the most effective for physically supporting the participant. But at the same time, in hand support, the delay between acceleration peaks from the participant and those from the facilitator was very short, and the two protagonists showed similar amounts of acceleration, which makes it very difficult to disentangle each protagonist's contribution. In the hand support, maybe more than in the two other support conditions, we can speak of the *co-production* of the messages by the participant and facilitator, and therefore of a *co-authorship* on the messages.

Third, in the forearm support condition, we observed that in 68% of cases, the acceleration peak of the participant preceded that of the facilitator, and that the facilitator did not produce any detected acceleration signals in almost 30% of cases. In this condition, the participant was also actively contributing to the typing process, either because he produced an acceleration motion first, or because he produced acceleration motions without any motor contribution from the facilitator at least at the index level. This result is therefore also likely warranting the participant's co-authorship on the messages. However, above the forearm support from the facilitator (holding the sleeve of the participant like a pendulum), it obviously does not rule out the possibility of other kinds of motor influence from his side, as well as from the participant's side, through subtle arm or forearm muscular micro-movements, which should be identified and measured in the future (e.g., using an electromyogram, despite its bad tolerance in people with moderate to severe forms of ASD). This further research could also allow for systematic characterization, quantification, and comparison of the movements exhibited by a participant in various typing conditions, in order to determine to what extent the various physical supports can attenuate his motor disturbances and enlarge his executive competencies.

Fourth, in the elbow support condition, the acceleration peak of the participant preceded that of the facilitator in 55% of cases, and the facilitator did not produce any detected acceleration signals in 43% of cases. Moreover, this elbow support condition revealed the largest difference in the amount of acceleration between the participant and facilitator (at the expense of the latter) compared to the two other support conditions. These findings reveal that the participant contributed even more to the messages co-production in this condition, i.e., this condition elicited his largest motor contribution to the typing process, and ensured his co-authorship on the messages.

Altogether, it is likely that the more proximal the physical support (i.e., closer to the hand of B.L.), the more B.L.'s motor disturbances (such as his lack of motor anticipation and initiative, repetitive or disorganized movements, perseveration, dyspraxia, and slowness, see Table 1) are supported and therefore attenuated by FC, as revealed by a faster speed of typing, a bigger motor contribution from the facilitator, and a similar amount of acceleration between the two protagonists. This parallel between proximal support and a higher level of help/facilitation on the motor disturbances of participants during FC was first observed by Crossley and Remington-Gurney (20). Indeed, motor peculiarities/disturbances and executive dysfunction are very frequent if not universal in individuals on the whole autism spectrum (21), and from the beginning of their life (22).

As said above, the physical support exerted by the facilitator during FC seems to filtrate and compensate these motor impairments. By what means? The physical support seemingly results in releasing a (larger or smaller) part of the weight of the arm/forearm of the patient, acting as a counter load or anti-gravity force, as it was suggested by Oudin et al. (23) using a mechanical device. The more proximal the physical support, the stronger the counter-load force exerted by the facilitator on the participant, the lesser the motor contribution (i.e., the physical effort) from the participant, and the faster the speed of typing.

Only very few studies to our knowledge have directly investigated the motor influence of the facilitator on participants with ASD. In one of these, Kesuka (24) using a strain gauge mechanical device with one participant with ASD, observed that time trials were slower as the length of ribbon increased (which is in line with our results), and concluded that the facilitator exerted an unconscious motor influence on the patient through subtle muscular movements. Unfortunately, no statistical analyses were available, which precludes any clear interpretation of the results. The author also acknowledged that “*the assistant and J* [the autistic patient] *seemed to move as unity in symbiosis; it was not a simple relationship of one partner acting on the other*” [p. 591]. This conclusion is supported by our results, especially those in hand support, and also raises the question of the other kinds of support involved in the FC process, such as psychic (emotional and motivational) support, which needs to be further explored.

As observable on Video, B.L. spent most time looking at the screen or at the keyboard in all the typing conditions, hence controlling visually the typing process, and confirming his own contribution to the message production. However, in a preliminary study (25) including B.L. and five other adults with ASD and intellectual disabilities, it was observed that far less time was spent looking at the screen or at the keyboard during the FC process in all the participants compared to B.L. in the present study. This intriguing phenomenon should be further studied in various contexts (natural vs. experimental; education/learning) in order to delineate various forms of facilitated communication, i.e., requiring more or less proximal holding from the facilitator, and requiring more or less visual and motor control and more or less overt/covert competencies from the participant.

Finally, this single case study should be replicated in well-diagnosed and well-assessed participants with ASD and/or other neurodevelopmental disorders (e.g., executive and motor disorders, verbal/oral communication impairments, etc.) using accelerometry as a control procedure, to test its reproducibility and validity.

Accelerometry is a non-invasive and relatively simple technique providing an accurate measurement of the course of some motor events involved in letters and word production or *co-production* during typing, and their variations according to the nature of physical support.

Finally, this single case of participant authorship (in the solo typing condition), or *co-authorship* (in the three support conditions) on messages produced *via* FC might hopefully contribute to revive the interest of clinicians and researchers in FC for the future.

## Data Availability Statement

All datasets generated for this study are included in the article/Supplementary Material.

## Ethics Statement

The study involving a human participant was reviewed and approved by Comité d'éthique d'Aix-Marseille université. Written informed consent to participate in this study was provided by the participants' legal guardian/next of kin. Written informed consent was obtained from the individual(s), and minor(s)' legal guardian/next of kin, for the publication of any potentially identifiable images or data included in this article.

## Author Contributions

BG, PF, and TL designed the study. BG diagnosed, assessed, and included the participant in the study. PF conducted the experiment. TL provided technical assistance and conducted the statistical analyses. BG wrote the first draft of the manuscript. BG, PF, and TL corrected and revised the manuscript and approved it in its final form. All authors contributed to the article and approved the submitted version.

## Conflict of Interest

The authors declare that the research was conducted in the absence of any commercial or financial relationships that could be construed as a potential conflict of interest.
